# How Could Children’s Storybooks Promote Empathy? A Conceptual Framework Based on Developmental Psychology and Literary Theory

**DOI:** 10.3389/fpsyg.2019.00121

**Published:** 2019-02-05

**Authors:** Natalia Kucirkova

**Affiliations:** ^1^Institute of Education, University College London, London, United Kingdom; ^2^Norwegian Centre for Learning Environment and Behavioural Research in Education, University of Stavanger, Stavanger, Norway

**Keywords:** empathy, books, stories, theory of mind, perspective-taking, literary theory, developmental psychology, social cognition

## Abstract

This conceptual paper proposes a framework for understanding the developmental mechanisms and literary characteristics that bind children’s storybooks with empathy. The article begins with a taxonomy of empathy composed of three key *continuous* dimensions: cognitive/emotional empathy, empathy for in-group and out-group members and empathy with positive and negative consequences. Insights from developmental psychology and literary theory form the basis for an interdisciplinary framework based on three premises: (1) book-reading can support empathy if it fosters in-group/out-group identification and minimizes in-group/out-group bias; (2) identification with characters who are dissimilar from the readers is the most valuable contribution of children’s storybooks to cognitive empathy; and (3) the quality of language positions children’s storybooks as an exceptional, but not exclusive, empathy-building form of fictional narratives. Implications for future intervention and empirical work are provided.

## Introduction

Empathy has been heralded as the key remedy to solve the disparities between major divisions in the currently interconnected globalized world (Krznaric, 2014), including countering toxic masculinity (Zimbardo, 2017) or the isolation and jealousy created by social media (Borba, 2016). As part of this encompassing role for empathy, there is an increasingly popular view among several authors, publishers, educators and literacy organizations, that storybook reading is the primary strategy to nurture children’s empathy skills. Slogans such as “Books build empathy” and initiatives such as the annual Empathy Day celebrated in United Kingdom schools, illustrate the enthusiasm of using children’s literature to promote empathy with “empathy-building books.” There is solid, undisputable evidence on the importance of book-reading for children’s language and literacy development (see Horst and Houston-Price, 2015 for a review). Hence, the question is not whether but *how* could children’s story-books be used in schools to promote additional outcomes, including empathy. Progress in answering this question has been made by several disciplines but mostly by developmental psychologists and literary critics. Yet, their joint contribution to the fundamental interactions between children’s reading of storybooks and empathy development is not reflected in current empirical and practical approaches. This paper addresses the current precision, conceptual and empirical inconsistencies.

### Precision Inconsistencies

Despite the frequent use of the word ‘empathy’ in common parlance, there are ongoing scholarly arguments about what empathy involves, how it can be operationalized and fostered in typical and atypical populations (see Sopcak et al., 2016). The word empathy is used to describe a set of different skills in different fields. Educationalists use empathy synonymously with several socio-emotional skills, but developmental psychologists understand these as distinct skills: theory of mind, perspective-taking, emotional literacies and emotional intelligences. Although we might treat these empathy-related phenomena as a conceptual cluster for convenience of expression, lumping them under one umbrella term of empathy creates several conceptual difficulties (see Decety and Cowell, 2014). For example, in social work education, there seems to be a perception that empathy is always a positive capacity (Grant, 2014). Yet, from a developmental perspective, theory of mind and perspective-taking have both positive and negative sides: enhanced theory of mind skills do not ‘simply, directly and inevitably translate into appropriate social behaviours.’ (Wellman, 2014, p. 61), because children’s enhanced theory of mind can also lead to anti-social behaviors such as lying and bullying. Furthermore, neurological studies and studies with specific populations of children, such as those on the autism spectrum disorder, show that some people have impaired affective but intact cognitive empathy and there is a significant qualitative difference between cognitive and emotional empathy skills in typically and atypically developing children (Baron-Cohen and Wheelwright, 2004). A meaningful application in specific contexts, such as in children’s reading, therefore requires a clear *specification of which type of empathy* might be promoted by books.

The second conceptual issue concerns the distinction between empathy for in-group and out-group members. In the context of adults’ empathy, Bloom (2017) argues that treating empathy as a moral guide is wrong because it perpetuates an in-group favoritism. Instead, he suggests rational compassion as a mechanism for understanding the mental states of out-group members. For literary theorists, Bloom’s argument does not accord with the fictional nature of stories and the diverse and abstract realities they portray. For developmental psychologists, there is a body of work on the positive and negative consequences of empathy. The *distinction between in-group and out-group facets of empathy* is thus essential for discussion of moral consequences of reading fictional texts in both adult *and* children’s populations.

Given the important social and moral role assigned to empathy, it is important to agree on a taxonomy of empathy that consolidates its cognitive and affective components as well as its positive and negative consequences for in- and out-group members. The first research question of this paper therefore is: How can empathy be conceptualized in relation to children’s storybooks? The conceptualisation incorporates insights from developmental psychology and literary theory, which have followed parallel but independent paths in the literature so far.

### Conceptual Inconsistencies

Conceptual precision is a sine qua non for empirical evaluations. The purported causal relationship between book reading and empathy is in popular media typically justified with the experimental evidence by Kidd and Castano (2013), who showed that reading literary fiction was associated with adults’ higher performance scores on theory of mind tasks, in comparison with reading non-fiction or popular fiction. However, the experiment was with *adult* readers and focused on theory of mind, which is one aspect, but not the only aspect, of empathy. Kidd and Castano (2013) were cautious about over-generalizations, caveating that while literary fiction might promote one type of emotional understanding, popular fiction and non-fiction might contribute differently to empathy. Moreover, motivational factors are essential constituents in readers’ short- and long-term engagement in reading and there are many psychology studies that point to the relationships between motivation, reading and empathy (e.g., Zaki, 2014).

The use of storybooks as empathy-building vehicles therefore needs to be considered in light of their specific affordances and the motivational catalysts that contribute to empathy-related outcomes. The second research question takes into account empirical evidence from developmental psychology and literary studies to ask: What are the mechanisms to support empathy through children’s storybooks?

### Empirical Deficiencies

Children’s books are considered an essential vehicle to discuss emotions and feelings, yet, empirical studies on the relationship between empathy and reading have been almost entirely preoccupied with adult readers and correlational. Research to date with adult readers shows that reading literary prose/literary fiction is related to readers’ ability to understand others (e.g., Mar et al., 2006). Crucially, recent research has shown that reading literary fiction in the digital format diminishes this effect (Mangen and Kuiken, 2014). Given that the studies are correlational, it is important to note the opposite direction of the presumed relationship, namely evidence that shows that people who are more empathic are more attracted to fiction (see Mar, 2018).

A body of experimental research with children shows that books can teach children cognitive skills, such as expressive and receptive language (e.g., Mendelsohn et al., 2001), or problem-solving and communication (e.g., Murray and Egan, 2014). Extant experimental research on children’s *digital* books is by and large preoccupied with cognitive outcomes, such as vocabulary learning and story comprehension (e.g., Bus et al., 2015; Dore et al., 2018) or qualitative explorations of parent–child dynamics during book reading (e.g., Chaudron et al., 2015). However, there is scarce research (cf. Kumschick et al., 2014) to support the view propagated by many best-selling children’s authors that their books “teach children empathy.” The specific features and mechanisms involved in storybook reading are multiple but this does not justify generic interventions, which are currently used to teach children empathy through commercially produced stories, profit-making programs and professional gate-keeping.

The third research question aims to trace the key factors identified by literary theory and developmental psychology in relation to storybooks and empathy and asks: which characteristics make children’s books a unique context for supporting children’s empathy development? Answering this question aims to provide a balanced view on the value of storybooks to foster children’s empathy in relation to other popular forms of narratives for children (e.g., educational films and video games).

## This Article

Against the backdrop of a conceptual and operational controversy concerning empathy, this paper aims to offer a nuanced and research-informed view on what early reading of storybooks might offer for children’s empathy. The theorized contribution of children’s developmental trajectory and literary techniques is mobilized to formulate a conceptual framework for empathy-building through children’s storybooks (EBCS for short). The review of studies focuses on children aged between two to eight because there is not enough scope to discuss age-related differences in empathy-related skills across a wider age span. The term *reading* is used in the broadest sense and interchangeably with *story engagement* to convey that even if young children are not proficient at decoding letters they derive meaning and pleasure from stories with the help of adult readers and this has an impact on their empathy-related skills. The focus is on children’s reading of *storybooks*, which are fictional narratives arranged in the form of a printed and bound text, structured as a beginning, middle and an end in a coherent structure governed by causality and temporality rules.

Storybooks can be prefaced with different nouns and adjectives and become specific categories of fictional narratives with distinct attributes. For example, *picture* storybooks are studied by literary scholars (e.g., Agosto, 1999; Nikolajeva and Scott, 2013) and *interactive* storybooks are studied by researchers in media studies (e.g., Adam and Wild, 1997). Nikolajeva (2014a) has discussed the role of aesthetic synergy for image/text, brain laterality and emotional literacy and Mangen (2008) has theorized the role of the digital format in influencing adults’ empathy in reading literary fiction. Due to space restrictions and the complexity of arguments involved in how different modalities relate to authors’ stylistic choices and how these might influence brain activity and emotional responses, the conceptual framework is limited to *text-based* storybooks and narratives represented in *written words*.

The third scope limitation relates to the specific fields of literary theory and developmental psychology rather than the study of children’s literature more widely and all divisions and sub-divisions of psychology. The different empirical focus and theoretical perspectives of literary theory and developmental psychology could be particularly complementary in identifying the role of storybooks in children’s empathy, which accords with calls for interdisciplinary approaches to studying children’s contemporary reading experiences (see Mangen and Van der Weel, 2016).

The study of literary theory and cognitive poetics covers a wide range of children’s books and involves an extensive quality analysis of the books’ features (note that children’s literature scholarship also includes children’s responses to books, using reader-response theory, social semiotics and other related disciplines). In contrast, developmental psychologists study the parent–child language *around* a book and they focus on children’s direct engagement with the book’s features. The EBCS framework includes insights from literary theory and developmental psychology because knowledge from the two disciplines is essential to realize the full potential of children’s reading and yet, the two disciplines tend to be represented by separate research communities that employ different research methods and rarely interact in joint publications or cross-disciplinary journals.

The article is structured as follows: the first section tackles the difficulties inherent in generic descriptors and inconsistent nomenclatures related to empathy. Drawing on developmental psychology, the developmental and socio-cultural basis for conceptualizing empathy in the context of children’s reading is introduced. The conceptual nuances of literary characteristics of children’s storybooks are combined with the terms used by developmental psychologists in describing the specific skills and abilities linked to empathy. The joint contribution of the disciplines results in a more precise conceptualisation, which forms the basis of the EBCS framework.

The second section provides the theoretical and analytical considerations necessary for establishing the mechanisms between children’s storybooks and empathy. Insights from literary theory and developmental psychology are synthesized to formulate a theory of change model which underpins empathy-building through children’s storybooks.

In the final section, the key empirical evidence available in literary theory and developmental psychology literatures is brought together for evaluating the potentially unique role of storybooks in fostering children’s empathy. The three sections build three premises of the EBCS conceptual framework, illustrated in three graphs.

## Conceptualization of Empathy in Early Childhood

### The Multifaceted Nature of Empathy

Empathy is known to be ‘a complex phenomenon that involves different intergroup, interpersonal and intrapersonal processes and mechanisms’ (Bertrand et al., 2018, n.d). The multidimensionality of empathy creates a fertile ground for its interdisciplinary study: empathy is studied and richly theorized in natural sciences as well as humanities and a single definition cannot cover the varied conceptualizations across various schools of thought.

Literary theory does not readily provide the vocabulary necessary for exacting the skills, abilities and dispositions involved in empathy-related phenomena. This section therefore draws primarily on developmental psychology to establish the specific facets of empathy related to children’s storybooks. Readers interested in how other psychology-related fields define empathy might find it useful to consult the reviews by Davis (1994) in social psychology, Decety and Jackson (2004) in behavioral and neurological sciences, Gallese (2001) in neuroscience, Cialdini et al. (1997) in personality psychology, White (1997) in clinical nursing, Beven et al. (2004) in forensic and legal psychology and Gilbert (2005) in relation to compassion in psychotherapy.

### The Developmental Trajectory of Empathy

Neurological and medical aspects of empathy suggest that empathy involves a recognition of other person’s feelings and a response to it, that is understanding another person’s mental state *and* acting on this understanding (Baron-Cohen, 2011). The recognition and response are both cognitive and emotional (Hoffman, 2001; Perry and Shamay-Tsoory, 2013). Cognitive empathy is the ‘capacity to engage in the cognitive process of adopting another person’s psychological point of view’ (ibid, p. 180), while emotional empathy involves ‘emotional contagion, emotional recognition, and shared pain’ (ibid, p. 179). From a neuroscientific perspective, the functional mechanisms of human cognition provide the foundation for a shared space of a self-other identity, which is bridged by mirror neurons (frontoparietal mirror-neuron areas, see Uddin et al., 2007), which, through the recognition of others’ actions, support self-representation. From Gallese’s (2005) neuroscientific perspective, mirror neurons instantiate a shared space that ‘blends the interacting individuals’ (p. 111) and provide a multimodal representation of organism-object relations. Mirror neurons, however, have been discovered and studied in adult populations. Developmental psychologists study the extent to which emotional and cognitive empathy are innate or can be nurtured in young children. There is a field consensus that emotional empathy appears earlier than cognitive empathy in a child’s developmental trajectory. For example, an early precursor to empathy is infants’ reactive cry: infants cry when they hear other infants crying and this cry is different from the cry when they are in discomfort (see Sagi and Hoffman, 1976). However, the ability to understand what others might think and use this information to, for example, intentionally deceive them, is a different cognitive skill. Cognitive empathy requires a child’s understanding of the beliefs and intentions of other people. In a very simplified way, we could consider the affective type of empathy to be more implicit and innate and the cognitive type more explicit and effortful because it requires the ability to mentalize, which children typically acquire between the age of three and four (Fonagy, 2018). Correspondingly, some disorders can be selectively related to either cognitive or affective empathy. Children with autism for example, are considered to have impaired cognitive empathy (Frith et al., 1991).

Cognitive empathy is synonymous with interpersonal empathy and *social cognition*, which refer to the human ability to mentalize and understand what other people think. In developmental psychology, social cognition involves the study of children’s theory of mind and perspective-taking. Social cognition requires more cognitive resources than emotional empathy (Saxe, 2006) and its development can be supported with specific techniques, strategies and resources. Therefore, from the developmental psychology perspective, if there is a relationship between children’s books and empathy, then it is more likely to be related to its cognitive variant. Given the focus on children’s storybooks in this article, cognitive empathy in the EBCS conceptual framework is foregrounded, acknowledging that children derive significant pleasure and enjoyment from reading their favorite stories.

### The Two Key Components of Cognitive Empathy

#### Perspective-Taking

Psychologists and clinicians use the term perspective-taking to describe children’s ability to differentiate between others’ and children’s own perspectives and to mentally imagine themselves into the shoes of someone else (Newman, 1986). The acquisition of perspective-taking skills has been observed in children around the age of three and four (see Wellman, 1992). Perspective-taking is an important research area in social psychology, where it is considered to encompass the ability to reason about how others perceive and understand the world *and* using this understanding to adjust one’s own view of others. Perspective-taking is thus not only about understanding but also actively overcoming one’s preconceived ideas, which can include biases and stereotypes (see Galinsky and Moskowitz, 2000) and inhabiting self-oriented (egocentric) views (Epley et al., 2004).

Although perspective-taking is well-known in lifespan theories of self-other differentiation, the term ‘decentering’ is specific to early childhood studies and was first extensively theorized by Jean Piaget. Montangero and Maurice-Naville (2013, p. 97) explain that according to Piaget, decentering is a ‘process of progressive dissociation or coordination’ and is tightly linked with children’s actions that start from concretization of ideas and progress to comparison and coordination. Both perspective-taking and decentering are about a child’s de-association from a subjective point of view. Redmond (1983) suggested that decentering would be a more encompassing term than perspective-taking but since 1980s, decentering has been mostly used in relation to Piaget’s original conceptualization.

#### Theory of Mind

Perspective-taking is different from, but closely related to, children’s theory of mind (ToM). First coined by Premack and Woodruff (1978) in their study with chimpanzees, ToM is a social ability to integrate into a coherent understanding what other people desire, believe or think (Gopnik et al., 1997). Early ToM studies focused on children’s understanding of false beliefs and investigating whether toddlers can recognize that other people have intentions different from theirs (see Carpendale and Chandler, 1996). More recent ToM studies combine neuro-imagining data with children’s accounts and show that there are networks of brain regions specifically related to ToM. For example, Saxe and Kanwishera (2003) showed that the temporo-parietal junction becomes activated when adults think about the mental state of other people. Language development goes hand in hand with empathy development and indeed, children’s ability to express how they and other people feel is another precursor for ToM. Parent–child engagement in conversational turns is a significant predictor for children’s language development, with distinct neural patterns identified in children who engage in frequent and responsive conversations with their parents (Romeo et al., 2018).

The key point of distinction in the psychological explanation of cognitive empathy is the difference between empathy for in-group and out-group members. This difference relates to the physical/psychological and perceived/felt *distance between others and self*, and is essential for understanding the positive and negative outcomes related to empathy.

### In- and Out-Group Empathy

From an evolutionary perspective, people experience an innate ‘intersubjective sympathy’ for in-group members (Trevarthen, 1979). This vicarious, somatic experience of shared emotions has been observed in several species that live in small social groups and express homophily (see Preston and de Waal, 2002, for an overview). Sympathy is not empathy but from a behavioural standpoint, the in-group/out-group distinction is intercorrelated with the cognitive/affective intersection in empathy. Neurological data show that different brain regions are involved in empathy for ingroup members versus empathy for humankind more broadly (Mathur et al., 2010). An in-group preference or positive group identification can support group belonging (Brewer, 1979), status stability and legitimacy (Struch and Schwartz, 1989), but it can also lead to a bias toward out-group members (Cikara and Van Bavel, 2014). There are negative consequences for both in- and out-group biases and there is evidence that White ethnic 3–4-year olds favor their ingroup members and show prejudice toward members of minority ethnic groups, with a decline for some children across middle childhood (Aboud, 2008).

The scientific jury is out on why in-group/out-group biases occur. From a sociological perspective, the in-group/out-group relationship is a fluid and dynamic process of negotiation related to three aspects of social categorization: positive affect, affiliation and social desirability (Zaki, 2014). Empathy is thus part of an ongoing bi-directional communicative act: ‘our relationships influence our emotions, and our emotions reciprocally influence our relationships’ (p. 2, Saarni, 1999). From a developmental perspective, there is an interesting parallel between older adults’ and young children’s preference for contact with close family members as opposed to peripheral friends and family members at early and late stages of life (Hess et al., 2009). The lacuna of studies on lifelong development makes it difficult to establish direct links between children’s and adults’ capacity of perspective-taking, but longitudinal evidence suggests a modest negative relation between age and perspective-taking (Pratt et al., 1996), with loss in perspective-taking documented in the elderly.

### Premise 1 of the EBCS Framework

**FIGURE 1:**
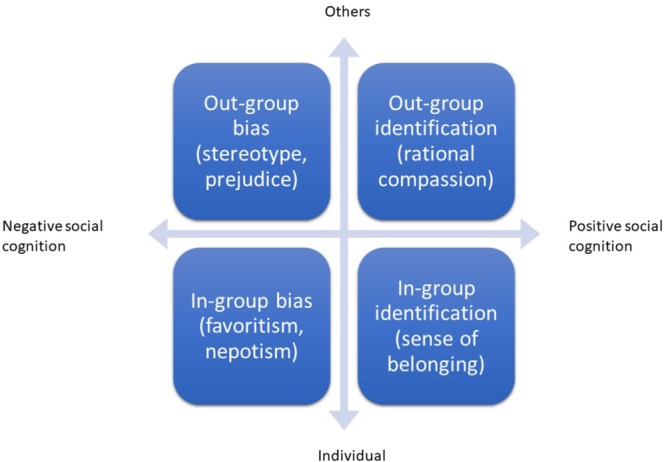
Types of identification and biases between readers and story protagonists.

If we synthesize the insights from decades of developmental psychology research on social cognition, we arrive at two dimensions that need to be incorporated into a conceptualisation of empathy-building with children’s storybooks: positive/negative valence of cognitive empathy and empathy toward in-group/out-group members. Children’s storybooks could potentially foster children’s *cognitive* empathy through a recognition and response toward in- *and* out-group members. The process of identification with others is a process influenced by group formation and social categorization, which leads to different outcomes, including group-belonging as well as bias. If we distil these tenets into a simple schema, we obtain a graph that identifies in-group/out-group and positive/negative outcomes of cognitive empathy. As Figure 1 shows, the *Y*-axis is the degree of affiliation with others (in-group versus out-group), while the *X*-axis represents the extent to which one’s social cognition is positive or negative. Their intersection creates four possible behavioral outcomes: when individuals identify with members of society who share their views/characteristics or experiences, they might experience a sense of belonging as well as in-group favoritism and out-group hostility. Conversely, if an individual mentalizes with out-group members without identifying with them, then that individual is likely to engage in what Bloom (2017) termed rational compassion. The graph illustrates that children’s books could potentially foster four possible pathways implicated in cognitive empathy.

This leads us to premise1 of the EBCS framework: book-reading can support children’s positive and negative cognitive empathy as it fosters both in-group/out-group identification *and* in-group/out-group bias. An immediate implication of this proposition is that children’s storybooks need to be written and designed to support both types of identification between the ‘other’ and ‘self,’ while avoiding their associated biases. Literary theorists and developmental psychologists could jointly contribute to greater awareness among popular children’s authors and organizations representing them, to ensure that children’s literature does not propagate in-group bias but actively fosters cognitive empathy toward out-group members.

Section I summarizes the theories and empirical data that underpin the key assumptions in the variables connected to cognitive empathy. Section II, that follows, discusses the mechanisms involved in the process that fosters positive outcomes of cognitive empathy with children’s storybooks.

If we have clear criteria of success and some data to predict patterns, we can progress to a prediction of effects. Such theoretical modeling is called a theory of change (Grant, 2014).

### Section II: Theory of Change: The Mechanisms Involved in Cognitive Empathy

In evaluation and performance monitoring, a theory of change is used to capture the process of how and why activities lead to desired outcomes. The concept of theory of change is used in this paper as a shorthand for explaining the potential pathways in which book reading could strengthen children’s cognitive empathy. Note that to discuss possible main and interaction effects, it is necessary to artificially separate the flows of individual components in the empathy process. The model is based on work by literary theorists and developmental psychologists concerning selective engagement in activities, the relationship between narrative fiction and empathy, and the role of personal relevance in story immersion and identification.

## Engagement and Motivation

### Selective Engagement

The neuropsychological and developmental literature agree that individuals respond to people based on the extent to which they feel close to them. There is one principle, Hess (2014) argues, that is applicable across the lifespan and possibly related to the in-group preference at the early and late stages of life: selective engagement. Hess’ (2006) Selective Engagement Hypothesis posits that individuals selectively allocate their cognitive and affective resources to engagements with others if the engagements offer a return of investment either in the form of increased knowledge (information-seeking) or emotion (affection-seeking behavior). This chimes with Wynn et al. (2017), who argue that children ‘naturally favor people whom they see as good individuals and who fall into their social groups’ (p. 2). The researchers describe babies and young children as ‘selective altruists’ and argue that the selective helping model in babies is propagated by three key developmental reasons: a child’s group belonging, past behavior and prior interactions with the other person (Wynn et al., 2017). A distinguishing contribution of the selective engagement hypothesis to the empathy discussion is that it introduces the importance of motivation in taking an action. If children are to learn empathy skills through books, then first and foremost, they need to be motivated to engage in reading the book.

### Motivation Catalysts

Motivation can be manipulated through various “catalysts” for empathy, which Bertrand et al. (2018) identified to be ‘emotionally safe environment, multicultural, collaborative, dynamic, engaging activities to stimulate openness, facilitators to support the learning process.’ Bertrand et al. (2018) include three methodologies that can train emphatic abilities: role-playing, mindfulness training and Enhancing Self-Regulation of Behavioral Expressions (e.g., Exhaustive practice of negation). Interestingly, the authors do not include literary fiction or written stories in their review but this omission might be attributable to their focus on virtual reality as a key mechanism for empathy training. We can think of Bertrand et al’s “catalysts for empathy” as the external and internal factors that contribute to a desired outcome. In a theory of change model, catalysts are called enablers and the outcomes are called outputs (Harries et al., 2014). In the EBCS model, the inputs are the cognitive and emotional resources, which a child employs to engage in book-reading. While the avoidance and regulatory motivation strategies involved in an emphatic response are widely known and studied in developmental psychology literature (e.g., Feldman, 2015; Doenyas, 2017), literary theorists place an emphasis on the literary characteristics of books involved in eliciting an emphatic response in readers. For literary theorists, there are the two key explanatory pathways for the relationship between storybooks and empathy: fiction and narratives.

## Fiction and Narratives

### Narratives

Narratives are a principal organizational structure for human thinking (Bruner, 1991). When paying attention to narratives, people tend to follow the experience of the protagonist, whether the narrative is represented in a written, pictorial or oral mode, thus directly practicing perspective-taking (Black et al., 1979). Mar and Oatley (2008) reviewed adults’ experience with narrative fiction in various formats, including novels, films and plays, and theorized that the experience is similar to a cognitive and emotional simulation of social experience and therefore might improve perspective-taking abilities. They argue that while the form/format or the fictional/non-fictional nature of stories are important considerations, it is *narrative* that is fundamental for perspective-taking.

### Fiction

The seminal work by Nikolajeva on children’s reading of fiction (Nikolajeva, 2009, 2014a,b) highlights the *fictional* nature of narratives written for young children. Given that in storybooks, the characters represent people or, in the case of children’s books, sometimes personified animals, their experiences simulate social experiences. These simulated, abstract experiences allow readers, including young readers, practicing their awareness of what people in different situations might feel or experience.

What is interesting to note is the different research priorities and the different direction of travel between empathy and text, identified by literary theorists and developmental psychologists. According to Maine and Waller (2011), empathy acts as a ‘tool of engagement’ in reading, while for psychologists, there is an opposite direction of travel: motivation drives individuals to either engage or disengage with the perspectives and emotions of others (Zaki, 2014). This difference in emphasis is reflected in the explanatory pathways provided by the two disciplines for how storybooks could foster children’s empathy.

## Explanatory Pathways in Developmental Psychology

Developmental psychologists study the empathy-building potential of the environment in which children’s storybooks are read (i.e., language around the book), the books’ content (i.e., language inside the book) and they also look beyond the printed page (i.e., the books’ format). These distinct elements are in bidirectional inter-relationship to developmental variables and they jointly constitute a complex matrix of enablers and outputs. There are three distinct research contributions that bring cohesive insight into the EBCS framework: environmental catalysts, adult-child talk, and metacognitive language.

### Environmental Catalysts

Most children of pre- and early primary-school-age are introduced to storybooks at home or in formal learning environments (such as schools and kindergartens) which are key “catalysts” for creating the empathy-inviting environment that Bertrand et al. (2018) described. The family structure provides a natural environment for parent–child conversations about in-group members’ feelings and perspectives. In a classic developmental text, Dunn and Kendrick (1982) outline how parents nurture children’s theory of mind by discussing the feelings and beliefs of siblings. It should be noted that children are able to fully benefit from meta-language conversations only when they can construct a coherent view of self and organize their autobiographical memories. This is typically achieved between the ages of three to five (Thompson, 2000). In the school environment, adults’ facilitation of empathic education programs, which include discussions of emotions and personal experiences, can promote children’s understanding of different views and reduce bullying (Şahin, 2012). Children’s cognitive empathy can be also trained by directly talking to children about mental states, as demonstrated by Guajardo and Watson (2002) in relation to narrative discourse and theory of mind. The key characteristics of effective empathy-building school environments are provision of explicit prompts to think about other people’s perspectives and the adults’ mediation of children’s understanding through conversation and direct discussion of views, emotions and experiences.

### Adult-Child Talk During Storybook Reading

In the context of adult-child book reading, books act as a joint object of reference for conversation about story characters’ feelings and thoughts (Symons et al., 2005). Some children’s books are an important source of mental state information, but it is the mothers’ *use* of mental state talk that can have a positive impact on children’s theory of mind understanding (Ruffman et al., 2002). Although everyday conversations at home might offer many opportunities for discussing how other people feel and think, parent–child conversations around storybooks offer unique opportunity for parents to discuss mental states of people their children don’t know. During shared book-reading, parents ask their children, for example, how they think the story protagonists feel, why the character might have acted in a certain way or what the character might think will happen on the next page. Mothers use various emotional words (to enjoy, to be afraid) and their own empathy levels are linked to the amount of ability states (e.g., ‘you can do it’) that they refer to during book sharing (Rollo and Sulla, 2016). Children whose mothers used cognitive verbs such as think, believe, remember, know or understand, during book-reading showed higher understanding of mental states after the book reading session (Adrián et al., 2007). In these exchanges, mothers’ talk was contingent upon the child’s response, as the mothers typically elaborated on aspects that they presumed the child might have found unclear and provided a cognitive scaffolding for the child’s understanding of the story details. Children too, engage in talking more about mental states during book reading than during everyday conversations, especially if they are older than 3 years and can fully appreciate the different beliefs and feelings portrayed in picture books (Sabbagh and Callanan, 1998).

### Metacognitive Language

The content of children’s storybooks conveys the story protagonists’ emotional states through pictures and words. Dyer et al. (2000) analyzed the amount of mental state language in 45 children’s picture books for 3–4-year-olds and in 45 books for 5–6-year-olds and found that books for 5–6-year-olds had more varied and more frequent mental state references, mostly represented in words and through the use of irony but less so via pictures. Textual references to mental states of story characters provide explanatory anchors for mentalizing because they approximate to children the characters’ thinking and feelings. However, Peskin and Astington (2004) found that a higher number of metacognitive terms in books does not necessarily improve children’s understanding of mental terms. Although more metacognitive terms in a book were related to children’s more frequent use of such terms, it was the adults’ mediation that helped children *infer* character’s mental states from their visual representation in the book.

**FIGURE 2:**
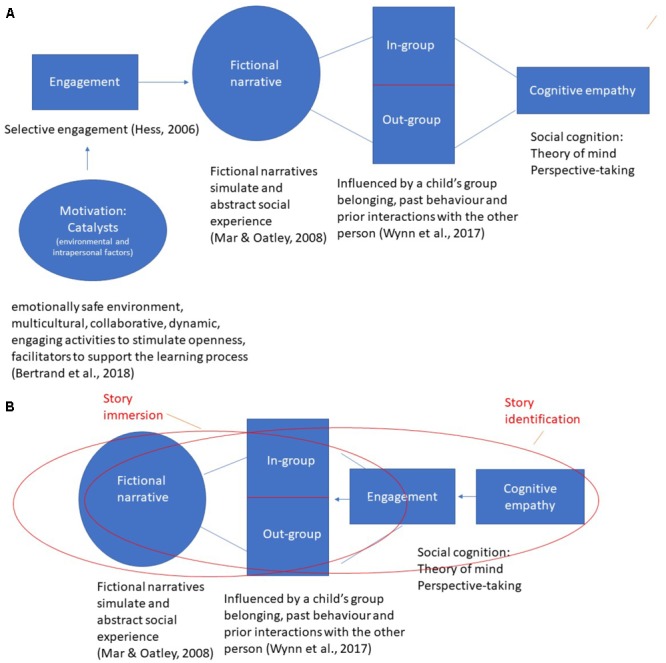
**(A)** Theory of change of reading and empathy emerging from developmental psychology. **(B)** Theory of change of reading and empathy emerging from the literary theory.

Thus, according to developmental psychology, some storybooks provide explicit prompts for discussing out-group members’ perspectives but the effects are likely to be increased if the book-reading is mediated by adults who engage in conversational turns with the child and scaffold the child’s understanding of diverse perspectives and emotions. This leads us to a theory of change model that delineates how empathy is triggered, what it promotes and why it matters for children’s development. Figure 2A schematically captures this process.

## Explanatory Pathways in Literary Theory

Based on literary theory, we can infer that children’s books create optimal conditions for supporting children’s cognitive empathy if there is a skilful use of narrative techniques to enhance readers’ identification with the story protagonist(s) who are *unlike* them because they are fictional. The summary of the key insights from literary theory is limited to the aspects that add a unique dimension to developmental psychology’s perspective: literary techniques, story immersion and story identification.

### Literary Techniques in Fictional Narratives

Keen (2007) highlights the complex relationship between empathy and narrative techniques such as the use of first or third person or past and present tense. She posits that each technique has different consequences for empathy depending on the book’s topic and the reading situation. Composition techniques studied by literary theorists include the point of view that authors employ for recounting the story (first, second, or third person); the authors’ choice of tense (present, past, and future), originality of the topic and its depiction, authentic voice, use of humor or irony and others. All elements need to fit the purpose and theme of the story and be adjusted to the story representation. For example, the first person in a written story involves more persuasion of subjectivity than the first person employed in oral narratives (Nielsen, 2004). The choice of present tense can enhance a story’s immediacy but it can also limit the reader’s imaginative landscape as ‘it confines the reader’s vicarious experience to a single consciousness in a temporal singularity’ (Nikolajeva, 2014a, p. 88).

### Story Immersion

Put simply, story immersion refers to the feeling of presence in the story-world created by an author. In the context of video games, McMahan (2003) specifies that immersion is contingent upon the story plot, the context of the story or unfolding narrative. In the context of book-reading, authors who can achieve readers’ immersion are authors whose books have become ‘books that can’t be put down’ and books that stir a personal response. Story immersion is directly linked to the delight that readers derive from engaging with a narrative and feelings of positive engagement and flow (Douglas and Hargadon, 2000). There are several story-telling elements that authors need to carefully combine to achieve the desired immersion effect in their readers. There is no single formula for this effect, but there are some common elements that are relevant for reader engagement, albeit in different ways. These include authors’ judicious choice of one of the five options of story movements (linear, meandering, spiral, branching, and explosive story movement, see Truby, 2008) and choice of a suitable textual level (e.g., the textual level among the text, writer and reader or the textual levels among various texts, see Nikolajeva, 2015). Nikolajeva (2009) anticipates the use of present tense and first-person narrator to bring the protagonist and reader closer to each other, but cautions against what she terms ‘immersive identification’ with story characters, as this would impede the possibility for perspective-taking.

Naturally, not every book, even if carefully crafted, will resonate with every child. This is where story immersion intersects with another important motivation-related aspect of storybooks: readers’ identification with the story characters.

### Story Identification

Nikolajeva’s (2014b) key thesis is that fiction should offer readers stories that are beyond their real-life experiences. This departure from personal experience alerts motivation in reading and emphatic engagement. The main motivation to read fiction comes from the desire to experience something the readers have not experienced themselves and identifying with characters who are fictional, for example unfamiliar settings and situations in horror stories or fantasy, with unfamiliar characters, such as monsters, wizards, or superheroes. Nikolajeva’s specification acknowledges the need for a balance between two key aspects of empathy (cognitive/affective) and its two ideals (empathy toward in-group and out-group members). The story immersion/character identification is a useful way for thinking about the optimal equilibrium necessary for a reader’s engagement with the narrative and identification with a story protagonist. Taken together, insights from literary theory bring us to a slightly different theory of change model for children’s story books and empathy, captured in Figure 2B.

### Premise 2

With these insights, we can tentatively formulate a theory of change that accounts for the relationship between story immersion, story identification and in-group/out-group empathy promoted by children’s narrative fiction. We could assume that the relationship between immersion and story engagement is a positive correlation: the more a child is immersed in a story, the more they are engaged with the narrative, deriving pleasure and delight from the reading experience. However, if we bring in the developmental psychology literature to this, we can add that the increased engagement mobilizes the cognitive resources necessary for engaging in cognitive empathy and the social cognition necessary for understanding protagonists who are unlike us.

**FIGURE 3:**
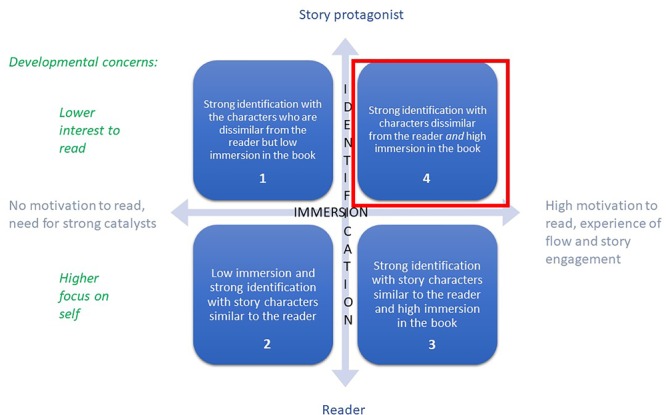
Schema of the strongest empathy-building potential of storybooks.

In Figure 3, the *X*-axis represents the story immersion spectrum (from no interest in the book to a high state of flow) and the *Y*-axis represents the perceived/felt distance between the reader and story protagonist. For illustrative purposes, the graph presupposes linearity between the variables so that the axes yield the borders of four quadrants that correspond to four possible outcomes. As indicated in the graph with the red square, Quadrant 4 illustrates the most desirable outcome: *children’s high immersion in the story and high identification with story characters who are unlike them*. As explained with the developmental trajectory literature, the cognitive resources that children need to employ to understand a protagonist who is different from their personal experience are higher than those required for identifying with a story character who shares their identity markers. Therefore, identification with characters dissimilar from the reader and the reader’s high immersion in the book requires most effort on the part of both the reader and the author. This outcome brings to fore the importance of literary craft in motivating children to read and using this motivation to foster the most resourceful type of empathy, that for out-group members.

Quadrant 4 also highlights that immersive stories do not necessarily create story characters that children can fully identify with. Quadrant 3 illustrates the corollary scenario where high story immersion is linked to high motivation to read, with feelings of flow and enjoyment of the story. However, unlike in the scenario depicted in Quadrant 4, higher immersion and higher personal relevance of the story character to the reader means empathizing with protagonists who share the readers’ characteristics (support of in-group cognitive empathy). Quadrant2 illustrates the case of personalized books where the story character *is* the child (e.g., the story hero is named after the child as it is the case in the personalized books by, for example, *I See Me Ltd.*). Quadrant 1 shows that strong identification with a story character (e.g., children seeing a popular character such as Peppa Pig in a book) can elicit their interest but combined with low immersion in the story, leads to higher need for environmental catalysts to support children’s interest in reading. Quadrants 1 and 2 demonstrate that low-quality books might engage children in reading but offer little to their cognitive empathy development.

The theory of change modeling leads to the second premise of the EBCS conceptual model: narrative fiction books are likely to promote children’s cognitive empathy for *in-group* members if they engage the child and if they feature story characters that children can readily identify with. The closer the personal relevance of the story to the child’s own life, the higher the likelihood for immersion, identification and in-group empathy. Conversely, children’s books are likely to promote children’s cognitive empathy for *out-group* members if they engage the child and at the same time, if they feature story protagonists with whom the readers can identify but who are dissimilar from their own lives. Literary theorists and developmental psychologists agree that a focus on out-group members is the most valuable characteristic of children’s storybooks, but while for literary theory the key motivational mechanism lies in the fictional nature of narratives, for developmental psychologists it is the role of adults’ mediation. These disciplinary nuances are crucial to attend to in building prediction models, evaluating various interventions and studying the relative influence of individual factors in research studies.

The focus on fictional narratives and story-related catalysts beckons the question: what is the specific role that children’s literature plays in cognitive empathy? In other words, there is a need to specify the catalysts (enablers) and the fictional nature of storybooks in relation to children’s engagement with narrative fictional storybooks. This conceptual specification is the goal of the third premise in the EBCS conceptual framework.

### Section III: The Unique Properties of Storybooks Relevant for Cognitive Empathy

According to Harries et al., 2014, a theory of change is effective if the activities in the model are based on unique qualities. Put more exactly, if we want to promote programs focused on *empathy-building skills with books*, then we need to be able to explain why empathy-building through books might be superior to other narrative representations. There are several types of narratives: oral storytelling, static and moving picture, audio stories, digital and written texts. Some fictional narrative contexts include a direct adoption of another person’s perspective, such as role-play, pretend play, drama and virtual reality. These fictional narrative contexts furnish the experience of perceiving the world from the perspective of another person, which, Mar and Oatley (2008) argue, might promote cognitive empathy. The mechanisms identified by literary theorists could therefore work for any narrative fiction. Similarly, the mechanisms identified by developmental psychologists, such as the presence of mental state language in narratives and its mediation by parents, could also occur in parent–child joint use of video games or watching educational TV.

Claims of superiority of one narrative context over another would require evidence of causality but for ethical and practical reasons, developmental psychologists cannot offer longitudinal evidence on the unique impact of storybooks in isolation from other narrative activities. Nevertheless, cross-sectional studies that examine the relative importance of one context against each other and match different media/representational modes according to specific criteria (e.g., the same narrative delivered on an iPad, via PC and in print), can offer some insights into how storybooks compare to other narrative contexts in relation to specific design or content features. The principal feature examined by extant experimental research concerned with different narrative contexts, is interactivity.

### Interactivity Across Contexts and Media

Comparative studies focused on children’s story comprehension and vocabulary learning in relation to different story formats and these studies converge that children’s learning is impeded by higher presence of interactivity (see e.g., Parish-Morris et al., 2013). The experimental studies suggest that interactivity interferes with children’s ability to focus and concentrate, which relates to recent neurological findings. Hutton et al., 2018 measured active imagery and self-reflection in pre-school children with functional magnetic resonance imaging in relation to picture-based, audio and interactive representation of the same story. They found that the illustrated format was more helpful and the animated format the least helpful for children’s self-reflection and active imagery. Based on these findings, we could tentatively assume that textual and static pictorial representation in children’s storybooks is conducive to children’s greater attention to the story. Whether this heightened focus can promote stronger perspective-taking and theory of mind is to be verified by empirical research. What is crucial, however, is the type of interactivity that might potentially interfere with children’s focus and attention to the story. However, literary theorists have proposed that some interactive features could be centrally implicated in children’s empathy-building with digital books.

### Types of Interactivity in Children’s Digital Storybooks

Zhao and Unsworth (2016) proposed that the opportunity to directly manipulate characters different from the child can contribute to children’s identification with them. They analyzed the specific features of a digital book (app) *The Heart and the Bottle*, and describe scenes where the user directly interacts with the story protagonist, which triggers a change in the thought bubble next to this character (e.g., moving the main protagonist across the screen changes the thought bubble from a happy memory of a grandfather to a sad memory of the grandfather’s departure). Drawing on Unsworth’s previous work, Zhao and Unsworth (2016) conclude that the app ‘constitutes an interpretative possibility (Unsworth, 2014a) of the story, a version of interpretation that creates ‘amplified empathy’ (Unsworth, 2014b; Zhao and Unsworth, 2016, p. 98).’ Similarly, Turrión (2014) and Al-Yaqout and Nikolajva (2015) argue that embodied engagement with story characters in digital interactive books, where readers replicate the actions of the main story characters, might stimulate children’s empathy.

The same text can be delivered digitally, in print or via moving image and each medium carries different interactive affordances that potentially support or interfere with empathy. The comparative studies to date have examined the influence of the format but *not the combined influence of the format and content* on child’s learning. Moreover, different types of interactivity could be potentially differently implicated in promoting children’s empathy through digital interactive books.

Kucirkova (2017) identified five types of interactivity in contemporary children’s fiction: synaesthesia (Touch manipulation, Audio presentation, Visual presentation, Taste properties, and Odor properties), Scaffolding (Audio, video-recorded or visual prompts, Hyperlinks to other explanatory content), Datafication (GPS tagging and usage tracking, Experience tracking), User control (Attention-directing features, Problem-solving features), and Computer vision techniques (Virtual-reality features such as action stimulator or environment creations, Augmented-reality features such as 3D representations of story characters or story plots). All these features need to be further theorized by literary theorists and experimentally studied by psychologists in different fictional narratives in relation to cognitive empathy.

### Individual Studies of Other Narrative Contexts

Based on the current literary theory and developmental psychology literatures, any fictional narrative, verbal, visual, or multimodal, has the potential to foster empathy. This implies that potentially, there are other narrative contexts that could foster children’s emphatic understanding and indeed, there is a wealth of research showing that other media that carry fictional narratives are related to children’s empathy.

More than 80 years ago, Mead (1934) identified children’s participation in role-and pretend play as a significant opportunity to practice reasoning and living as someone else. During pretend play, children can practice false belief and deception and these activities can supplement the use of books. There is also evidence from educational TV programs that empathy-related skills can be fostered through video content. For instance, Mares and Pan (2013) meta-analyzed the effects of the Sesame Street program on children in 15 countries and found a strong positive effect for the category labeled ‘social reasoning,’ which included the sub-category of children’s development of positive attitudes toward social out-groups. The researchers expected a small effect size given that young children’s development of positive attitudes toward visually salient differences (such as disability or race) is often difficult to nurture. The researchers explained that because the Sesame Street characters displayed prosocial behavior with out-group characters, this modeling had a significant effect on children’s perceptions.

Wolf (2018) cites studies with adult readers that show a correlation between reading fiction and cognitive processes that underlie perspective taking and theory of mind in support of the thesis that empathy occurs with high-quality written texts and literary novels. More direct evidence is needed on the specific types of texts and empathy processes in young readers, especially in light of alternative narrative formats and types of fiction. In particular, according to the embodiment perspective, virtually experiencing to be another person is superior than imagining it. It follows that children’s active participation in drama, role-play and pretend play could potentially constitute more effective contexts for empathy-building than storybooks. With adult participants, virtual reality games were found to support empathy because they allow full immersion into the protagonist’s perspective by embodying the protagonist’s body or virtually becoming another person through a digital avatar (Maselli and Slater, 2013). Again, there is no study that would investigate possible differences in the empathy-building potential of different interactive narrative representations such as virtual games, educational videos or digital stories. In the absence of empirical data showing superior effects for text-based storybooks for children’s cognitive empathy, it is difficult to know which format-related features are most effective. The content/format intersection thus offers fertile ground for examining new fictional narrative contexts and their empathy-related potential.

### Premise 3 of the EBCS Framework

Taken together, Premise 3 of the EBCS conceptual framework therefore is that children’s storybooks can act as prompts for practicing cognitive empathy if they represent fictional narratives with diverse and frequent references to story characters’ feelings and offer space for adults’ mediation. However, whether written texts (print books) constitute a superior narrative context in comparison to other fictional narrative contexts is not clear from the current literature.

## Study Implications

This paper combines multidisciplinary insights to arrive at a grounded framework that locates the key pathways and elements implicated in empathy and children’s written storybooks. The field has advanced in terms of recognizing the distinction, but also the connection, between affective and cognitive types of emphatic responses to books. There is enough empirical evidence to claim that books provide a context for practicing perspective-taking and identifying with the ‘other.’ It is time to move the field forward in relation to the possibly unique relationship between children’s storybooks and empathy and the specifics of the relationship between ‘self’ and the ‘other’ encountered through books. The EBCS framework specifies that not all storybooks promote positive cognitive empathy but those that do, challenge children cognitively and emotionally to understand the perspective of protagonists who are *unlike* them. The three premises and their operational implications make it clear that children’s storybooks could promote children’s understanding of others’ perspectives if there is a judicious balance between a set of requirements: cognitive/affective empathy, in-/out-group identification, story immersion/character identification, narrative representation in words and adults’ conversational mediation.

Although this premise is implicit in the works of literary theorists and developmental psychologists, it is sometimes not understood by popular children’s authors, who, often unwittingly, promote in-group bias by suggesting that to foster empathy, children should be asked about book characters who are most like them (see the Twitter chat transcript for #EmpathyDay, 10th of June, 2018). Although research has, thus far, not isolated a single factor that would make children’s storybooks a unique medium for empathy-building fictional narratives, there are some questions that educational professionals could be asking about empathy-building books in ascertaining their overall “empathy value.” For example, the EBCS framework could be developed into a rubric of empathy characteristics of children’s storybooks in collaboration with children’s publishers, designers, and developers. Examples of questions are:

Do the identity markers of the main protagonist correspond to those of the reader? [yes indicates lower empathy potential].

Does the storybook contain prompts for adults’ scaffolding and frequent use of meta-language with cognitive verbs (e.g., think, remember, and believe)? [higher score indicates higher empathy potential].

The three premises of the EBCS framework can be used for justifying the development of interventions and resources aimed at developing children’s empathy through storybooks. There are several interventions, programs and organizations that aim to support empathy, with various foci: for example, supporting children’s development of empathy-related behavioral outcomes, such as compassion and caring, through the care for vulnerable groups, such as babies (e.g., Gordon, 2000) or animals (Komorosky and O’Neal, 2015); through narrating and acting out children’s own stories (MakeBelieveArts) or promotion of films such as *To Kill a Mockingbird* or *Inside Out and Zootopia* (Common Sense Media). The EBCS framework specifies the conditions for gauging children’s empathy-building with books but it cautions against adopting an all-encompassing view of storybooks as the only vehicle for supporting children’s empathy. Future work needs to provide more clarity on the specific influence of the format of fictional narratives and the individual empathy-building features of children’s storybooks. This is important in an era of new story formats, which allow for easy manipulation of selected features, such as interactive or personalized storybooks. Organizations dedicated to promoting children’s empathy through storybooks, such as the United Kingdom-based EmpathyLab, could collaborate with researchers, authors, and educational professionals to ascertain the specific content and format affordances of children’s storybooks implicated in cognitive empathy.

Overall, the three premises of this interdisciplinary conceptual framework provide a necessary foundation to begin to answer empirically and practically the question of how children’s books could promote empathy. This paper argued and demonstrated that the joint work of literary theorists and developmental psychologists is essential to help educational practitioners and policy-makers make discerned decisions about investing in a specific intervention/approach dedicated to supporting children’s empathy. The framework can be applied to characterize the empathy concepts employed by different disciplines and stakeholders interested in nurturing children’s empathy with storybooks, to review earlier attempts and propose future empirical work in the area.

## Author Contributions

NK conceptualized the study and led the write-up of the paper.

## Conflict of Interest Statement

The author declares that the research was conducted in the absence of any commercial or financial relationships that could be construed as a potential conflict of interest.

## References

[B1] Aboud F. E. (2008). “A social-cognitive developmental theory of prejudice,” in *Handbook of Race, Racism, and the Developing Child* eds Quintana S. M. McKown C. (Hoboken, NJ: Wiley) 55–72.

[B2] Adam N. Wild M. (1997). Applying CD-ROM interactive storybooks to learning to read. *J. Comput. Assist. Learn.* 13 119–132. 10.1046/j.1365-2729.1997.00014.x

[B3] Adrián J. E. Clemente R. A. Villanueva L. (2007). Mothers’ use of cognitive state verbs in picture-book reading and the development of children’s understanding of mind: a longitudinal study. *Child Dev.* 78 1052–1067. 10.1111/j.1467-8624.2007.01052.x 17650125

[B4] Agosto D. E. (1999). One and inseparable: Interdependent storytelling in picture storybooks. *Child. Lit. Educ.* 30 267–280. 10.1023/A:1022471922077

[B5] Al-Yaqout G. Nikolajva M. (2015). Re-conceptualising picturebook theory in the digital age. *Barnelitterært Forskningstidsskrift* 6 269–271. 10.3402/blft.v6.26971

[B6] Baron-Cohen S. (2011). *Zero Degrees of Empathy: A New Theory of Human Cruelty* Vol. 30. London: Penguin.

[B7] Baron-Cohen S. Wheelwright S. (2004). The empathy quotient: an investigation of adults with Asperger syndrome or high functioning autism, and normal sex differences. *J. Autism Dev. Disord.* 34 163–175. 10.1023/B:JADD.0000022607.19833.00 15162935

[B8] Bertrand P. Guegan J. Robieux L. McCall C. A. Zenasni F. (2018). Learning empathy through virtual reality: multiple strategies for training empathy-related abilities using body ownership Illusions in embodied virtual reality. *Front. Robot. AI* 5:26 10.3389/frobt.2018.00026 PMC7805971 33500913

[B9] Beven J. P. O’Brien-Malone A. Hall G. (2004). Using the interpersonal reactivity index to assess empathy in violent offenders. *Int. J. Forensic Psychol.* 1 33–41.

[B10] Black J. B. Turner T. J. Bower G. H. (1979). Point of view in narrative comprehension, memory, and production. *J. Verb. Learn. Verb. Behav.* 18 187–198. 10.1016/S0022-5371(79)90118-X

[B11] Bloom P. (2017). *Against Empathy: The Case for Rational Compassion.* New York, NY: Random House.

[B12] Borba M. (2016). *UnSelfie: Why Empathetic Kids Succeed in Our All-About-Me World*: New York, NY: Simon and Schuster.

[B13] Brewer M. B. (1979). In-group bias in the minimal intergroup situation. *Psychol. Bull.* 86 307–324. 10.1037/0033-2909.86.2.307

[B14] Bruner J. (1991). The narrative construction of reality. *Crit. Inq.* 18 1–21. 10.1086/448619

[B15] Bus A. G. Takacs Z. K. Kegel C. A. (2015). Affordances and limitations of electronic storybooks for young children’s emergent literacy. *Dev. Rev.* 35 79–97. 10.1016/j.dr.2014.12.004

[B16] Carpendale J. I. Chandler M. J. (1996). On the distinction between false belief understanding and subscribing to an interpretive theory of mind. *Child Dev.* 67 1686–1706. 10.2307/1131725

[B17] Chaudron S. Beutel M. Donoso Navarrete V. Dreier M. Fletcher-Watson B. Heikkilä A. (2015). *Young Children (0-8) and Digital Technology: A Qualitative Exploratory Study Across Seven Countries.* Available at: http://publications.jrc.ec.europa.eu/repository/handle/JRC93239

[B18] Cialdini R. B. Brown S. L. Lewis B. P. Luce C. Neuberg S. L. (1997). Reinterpreting the empathy–altruism relationship: when one into one equals oneness. *J. Pers. Soc. Psychol.* 73 481–494. 10.1037/0022-3514.73.3.481 9294898

[B19] Cikara M. Van Bavel J. J. (2014). The neuroscience of intergroup relations: an integrative review. *Perspect. Psychol. Sci.* 9 245–274. 10.1177/1745691614527464 26173262

[B20] Davis M. H. (1994). *Empathy: A Social Psychological Approach.* London: Routledge.

[B21] Decety J. Cowell J. M. (2014). The complex relation between morality and empathy. *Trends Cogn. Sci.* 18 337–339. 10.1016/j.tics.2014.04.008 24972506

[B22] Decety J. Jackson P. L. (2004). The functional architecture of human empathy. *Behav. Cogn. Neurosci. Rev.* 3 71–100. 10.1177/1534582304267187 15537986

[B23] Doenyas C. (2017). Self versus other oriented social motivation, not lack of empathic or moral ability, explains behavioral outcomes in children with high theory of mind abilities. *Motiv. Emot.* 41 683–697. 10.1007/s11031-017-9636-4

[B24] Dore R. A. Hassinger-Das B. Brezack N. Valladares T. L. Paller A. Vu L. (2018). The parent advantage in fostering children’s e-book comprehension. *Early Child. Res. Q.* 44 24–33. 10.1016/j.ecresq.2018.02.002

[B25] Douglas Y. Hargadon A. (2000). The pleasure principle: immersion, engagement, flow. *Paper Presented at the Proceedings of the 11th ACM on Hypertext and Hypermedia* New York, NY 10.1145/336296.336354

[B26] Dunn J. Kendrick C. (1982). *Siblings: Love, Envy, & Understanding.* Cambridge, MA: Harvard University Press 10.4159/harvard.9780674330597

[B27] Dyer J. R. Shatz M. Wellman H. M. (2000). Young children’s storybooks as a source of mental state information. *Cogn. Dev.* 15 17–37. 10.1016/S0885-2014(00)00017-4

[B28] Epley N. Keysar B. Van Boven L. Gilovich T. (2004). Perspective taking as egocentric anchoring and adjustment. *J. Pers. Soc. Psychol.* 87 327–329. 10.1037/0022-3514.87.3.327 15382983

[B29] Feldman R. (2015). Mutual influences between child emotion regulation and parent–child reciprocity support development across the first 10 years of life: implications for developmental psychopathology. *Dev. Psychopathol.* 27(4 pt. 1) 1007–1023. 10.1017/S0954579415000656 26439059

[B30] Fonagy P. (2018). *Affect Regulation, Mentalization and the Development of the Self.* New York, NY: Routledge 10.4324/9780429471643

[B31] Frith U. Morton J. Leslie A. M. (1991). The cognitive basis of a biological disorder: autism. *Trends Neurosci.* 14 433–438. 10.1016/0166-2236(91)90041-R 1722361

[B32] Funnell S. C. Rogers P. J. (2011). *Purposeful Program Theory: Effective Use of Theories of Change and Logic Models (Vol. 31).* San Francisco, CA: John Wiley & Sons.

[B33] Galinsky A. D. Moskowitz G. B. (2000). Perspective-taking: decreasing stereotype expression, stereotype accessibility, and in-group favoritism. *J. Pers. Soc. Psychol.* 78 708–724. 10.1037/0022-3514.78.4.708 10794375

[B34] Gallese V. (2001). The shared manifold hypothesis. From mirror neurons to empathy. *J. Conscious. Stud.* 8 33–50. 14504450

[B35] Gallese V. (2005). Being like me”: self-other identity, mirror neurons and empathy. *Perspect. Imitat. Cogn. Neurosci. Soc. Sci.* 1 101–118.

[B36] Gilbert P. (2005). *Compassion: Conceptualisations, Research and Use in Psychotherapy.* New York, NY: Routledge.

[B37] Gopnik A. Meltzoff A. N. Bryant P. (1997). *Words, Thoughts, and Theories* Vol. 1 Cambridge, MA: MIT Press

[B38] Gordon M. (2000). *Roots of Empathy. Training Manual.* Toronto: The Maytree Foundation.

[B39] Grant L. (2014). Hearts and minds: aspects of empathy and wellbeing in social work students. *J. Soc. Work Educ.* 33 338–352. 10.1080/02615479.2013.805191

[B40] Guajardo N. R. Watson A. C. (2002). Narrative discourse and theory of mind development. *J. Genet. Psychol.* 163 305–325. 10.1080/00221320209598686 12230152

[B41] Harries E. Hodgson L. Noble J. (2014). *Creating Your Theory of Change: NPC’s Practical Guide.* Available at: http://www.thinknpc.org/publications/creating-your-theory-of-change

[B42] Hess T. M. (2006). Adaptive aspects of social cognitive functioning in adulthood: age–related goal and knowledge influences. *Soc. Cognit.* 24 279–309. 10.1521/soco.2006.24.3.279

[B43] Hess T. M. (2014). Selective engagement of cognitive resources: motivational influences on older adults’ cognitive functioning. *Perspect. Psychol. Sci.* 9 388–407. 10.1177/1745691614527465 26173272 PMC5911399

[B44] Hess T. M. Leclerc C. M. Swaim E. Weatherbee S. R. (2009). Aging and everyday judgments: the impact of motivational and processing resource factors. *Psychol. Aging* 24 735–740. 10.1037/a0016340 19739930 PMC2742956

[B45] Hoffman M. L. (2001). *Empathy and Moral Development: Implications for Caring and Justice.* Cambridge, MA: Cambridge University Press.

[B46] Horst J. S. Houston-Price C. (2015). An open book: what and how young children learn from picture and story books. *Front. Psychol.* 6:1719. 10.3389/fpsyg.2015.01719 26617549 PMC4639599

[B47] Hutton J. S. Dudley J. Horowitz-Kraus T. DeWitt T. Holland S. K. (2018). Differences in functional brain network connectivity during stories presented in audio, illustrated, and animated format in preschool-age children. *Brain Imaging Behav.* 1–12. 10.1007/s11682-018-9985-y 30377932

[B48] Keen S. (2007). *Empathy and the Novel.* Oxford: Oxford University Press on Demand 10.1093/acprof:oso/9780195175769.001.0001

[B49] Kidd D. C. Castano E. (2013). Reading literary fiction improves theory of mind. *Science* 342 377–380. 10.1126/science.1239918 24091705

[B50] Komorosky D. O’Neal K. K. (2015). The development of empathy and prosocial behavior through humane education, restorative justice, and animal-assisted programs. *Contemp. Justice Rev.* 18 395–406. 10.1080/10282580.2015.1093684

[B51] Krznaric R. (2014). *Empathy: A Handbook for Revolution.* London: Random House.

[B52] Kucirkova N. (2017). An integrative framework for studying, designing and conceptualising interactivity in children’s digital books. *Br. Educ. Res. J.* 43 1168–1185. 10.1002/berj.3317

[B53] Kumschick I. R. Beck L. Eid M. Witte G. Klann-Delius G. Heuser I. (2014). READING and FEELING: the effects of a literature-based intervention designed to increase emotional competence in second and third graders. *Front. Psychol.* 5:1448 10.3389/fpsyg.2014.01448 25566129 PMC4267422

[B54] Maine F. Waller A. (2011). Swallows and amazons forever: How adults and children engage in reading a classic text. *Child. Lit. Educ.* 42 354–371. 10.1007/s10583-011-9139-y

[B55] Mangen A. (2008). Hypertext fiction reading: haptics and immersion. *J. Res. Read.* 31 404–419. 10.1111/j.1467-9817.2008.00380.x

[B56] Mangen A. Kuiken D. (2014). Lost in an iPad: narrative engagement on paper and tablet. *Sci. Study Lit.* 4 150–177. 10.1075/ssol.4.2.02man

[B57] Mangen A. Van der Weel A. (2016). The evolution of reading in the age of digitisation: an integrative framework for reading research. *Literacy* 50 116–124. 10.1111/lit.12086

[B58] Mar R. A. (2018). Evaluating whether stories can promote social cognition: introducing the Social Processes and Content Entrained by Narrative (SPaCEN) framework. *Discourse Process.* 55 454–479. 10.1080/0163853X.2018.1448209

[B59] Mar R. A. Oatley K. (2008). The function of fiction is the abstraction and simulation of social experience. *Perspect. Psychol. Sci.* 3 173–192. 10.1111/j.1745-6924.2008.00073.x 26158934

[B60] Mar R. A. Oatley K. Hirsh J. de la Paz J. Peterson J. B. (2006). Bookworm versus nerds: exposure to fiction versus non-fiction, divergent associations with personality, ability, and achievement. *J. Res. Pers.* 40 694–712. 10.1016/j.jrp.2005.08.002

[B61] Mares M.-L. Pan Z. (2013). Effects of sesame street: a meta-analysis of children’s learning in 15 countries. *J. Appl. Dev. Psychol.* 34 140–151. 10.1016/j.appdev.2013.01.001

[B62] Maselli A. Slater M. (2013). The building blocks of the full body ownership illusion. *Front. Hum. Neurosci.* 7:83. 10.3389/fnhum.2013.00083 23519597 PMC3604638

[B63] Mathur V. A. Harada T. Lipke T. Chiao J. Y. (2010). Neural basis of extraordinary empathy and altruistic motivation. *Neuroimage* 51 1468–1475. 10.1016/j.neuroimage.2010.03.025 20302945

[B64] McMahan A. (2003). Immersion, engagement and presence. *Video Game Theory Read.* 67:86.

[B65] Mead G. H. (1934). *Mind, Self and Society* Vol. 111 Chicago, IL: University of Chicago Press

[B66] Mendelsohn A. L. Mogilner L. N. Dreyer B. P. Forman J. A. Weinstein S. C. Broderick M. (2001). The impact of a clinic-based literacy intervention on language development in inner-city preschool children. *Pediatrics* 107 130–134. 10.1542/peds.107.1.130 11134446

[B67] Montangero J. Maurice-Naville D. (2013). *Piaget or the advance of knowledge: An overview and glossary.* Mahwah, NJ: Psychology Press 10.4324/9780203763711

[B68] Murray A. Egan S. M. (2014). Does reading to infants benefit their cognitive development at 9-months-old? An investigation using a large birth cohort survey. *Child Lang. Teach. Ther.* 30 303–315. 10.1177/0265659013513813

[B69] Newman D. (1986). The role of mutual knowledge in the development of perspective taking. *Dev. Rev.* 6 122–145. 10.1016/0273-2297(86)90008-0

[B70] Nielsen H. S. (2004). The impersonal voice in first-person narrative fiction. *Narrative* 12 133–150. 10.1353/nar.2004.0002

[B71] Nikolajeva M. (2009). *Power, Voice and Subjectivity in Literature for Young Readers.* New York, NY: Routledge 10.4324/9780203866924

[B72] Nikolajeva M. (2014a). Memory of the present: empathy and identity in young adult fiction. *Narrat. Works* 4 86–107.

[B73] Nikolajeva M. (2014b). *Reading for Learning: Cognitive Approaches to Children’s Literature* Vol. 3. Amsterdam: John Benjamins Publishing Company 10.1075/clcc.3

[B74] Nikolajeva M. (2015). *Children’s Literature Comes of Age: Toward a New Aesthetic.* London: Routledge 10.4324/9781315667492

[B75] Nikolajeva M. Scott C. (2013). *How Picturebooks Work.* New York, NY: Routledge 10.4324/9780203960615

[B76] Parish-Morris J. Mahajan N. Hirsh-Pasek K. Golinkoff R. M. Collins M. F. (2013). Once upon a time: parent–child dialogue and storybook reading in the electronic era. *Mind Brain Educ.* 7 200–211. 10.1111/mbe.12028

[B77] Perry A. Shamay-Tsoory S. (2013). Understanding emotional and cognitive empathy: a neuropsychological. in *Understanding Other Minds: Perspectives From Developmental Social Neuroscience* eds Baron-Cohen H. T.-F. S. Lombardo M. (OUP: Oxford) 178–195. 10.1093/acprof:oso/9780199692972.003.0011

[B78] Peskin J. Astington J. W. (2004). The effects of adding metacognitive language to story texts. *Cogn. Dev.* 19 253–273. 10.1016/j.cogdev.2004.01.003

[B79] Pratt M. W. Diessner R. Pratt A. Hunsberger B. Pancer S. M. (1996). Moral and social reasoning and perspective taking in later life: a longitudinal study. *Psychol. Aging* 11 66–73. 10.1037/0882-7974.11.1.66 8726371

[B80] Premack D. Woodruff G. (1978). Does the chimpanzee have a theory of mind? *Behav. Brain Sci*. 1 515–526. 10.1017/S0140525X00076512

[B81] Preston S. D. de Waal F. B. (2002). Empathy: its ultimate and proximate bases. *Behav. Brain Sci.* 25 1–20. 12625087 10.1017/s0140525x02000018

[B82] Redmond M. V. (1983). Towards resolution of the confusion among the concepts empathy, role-taking, perspective taking, and decentering. *Paper Presented at the Annual Meeting of the 69th Speech Communication Association* Washington, DC.

[B83] Rollo D. Sulla F. (2016). Maternal talk in cognitive development: relations between psychological lexicon, semantic development, empathy, and temperament. *Front. Psychol.* 7:394. 10.3389/fpsyg.2016.00394 27047421 PMC4801865

[B84] Romeo R. R. Leonard J. A. Robinson S. T. West M. R. Mackey A. P. Rowe M. L. (2018). Beyond the 30-Million-word gap: children’s conversational exposure is associated with language-related brain function. *Psychol. Sci.* 29:700–710. 10.1177/0956797617742725 29442613 PMC5945324

[B85] Ruffman T. Slade L. Crowe E. (2002). The relation between children’s and mothers’ mental state language and theory-of-mind understanding. *Child Dev.* 73 734–751. 10.1111/1467-8624.00435 12038548

[B86] Saarni C. (1999). *The Development of Emotional Competence.* New York, NY: Guilford Press.

[B87] Sabbagh M. A. Callanan M. A. (1998). Metarepresentation in action: 3-, 4-, and 5-year-olds’ developing theories of mind in parent–child conversations. *Dev. Psychol.* 34 491–502. 10.1037/0012-1649.34.3.491 9597359

[B88] Sagi A. Hoffman M. L. (1976). Empathic distress in the newborn. *Dev. Psychol.* 12 175–176. 10.1037/0012-1649.12.2.175

[B89] Şahin M. (2012). An investigation into the efficiency of empathy training program on preventing bullying in primary schools. *Child. Youth Serv. Rev.* 34 1325–1330. 10.1016/j.childyouth.2012.03.013

[B90] Saxe R. (2006). Uniquely human social cognition. *Curr. Opin. Neurobiol.* 16 235–239. 10.1016/j.conb.2006.03.001 16546372

[B91] Saxe R. Kanwishera N. (2003). People thinking about thinking people the role of the temporo-parietal junction in “theory of mind.” *Neuroimage* 19 1835–1842. 10.1016/S1053-8119(03)00230-1 12948738

[B92] Sopcak P. Salgaro M. Herrmann J. B. (2016). Transdisciplinary approaches to literature and empathy. *Sci. Study Lit.* 6 2–5. 10.1075/ssol.6.1.02sop

[B93] Struch N. Schwartz S. H. (1989). Intergroup aggression: Its predictors and distinctness from in-group bias. *J. Pers. Soc. Psychol.* 56 364–373. 10.1037/0022-3514.56.3.364 2926634

[B94] Symons D. K. Peterson C. C. Slaughter V. Roche J. Doyle E. (2005). Theory of mind and mental state discourse during book reading and story-telling tasks. *Br. J. Dev. Psychol.* 23 81–102. 10.1348/026151004X21080

[B95] Thompson R. A. (2000). The legacy of early attachments. *Child Dev.* 71 145–152. 10.1111/1467-8624.00128 10836568

[B96] Trevarthen C. (1979). “Communication and cooperation in early infancy: a description of primary intersubjectivity,” in *Before Speech: The Beginning of Interpersonal Communication* Vol. 1 ed. Bullowa M. (New York, NY: Cambridge University Press) 321–347.

[B97] Truby J. (2008). *The Anatomy of Story: 22 Steps to Becoming a Master Storyteller.* New York, NY: Farrar, Straus and Giroux.

[B98] Turrión C. (2014). Multimedia book apps in a contemporary culture: commerce and innovation, continuity and rupture. *Barnelitterært Forskningstidsskrift* 5:24426 10.3402/blft.v5.24426

[B99] Uddin L. Q. Iacoboni M. Lange C. Keenan J. P. (2007). The self and social cognition: the role of cortical midline structures and mirror neurons. *Trends Cogn. Sci.* 11 153–157. 10.1016/j.tics.2007.01.001 17300981

[B100] Unsworth L. (2014a). *Interfacing Visual and Verbal Narrative art in Paper and Digital Media: Recontextualising Literature and Literacies. In Literacy in the Arts.* Berlin: Springer 55–76. 10.1007/978-3-319-04846-8_4

[B101] Unsworth L. (2014b). “Point of view in picture books and animated film adaptations: Informing critical multimodal comprehension and composition pedagogy,” in *Critical Multimodal Studies of Popular Culture* eds Djonov E. Zhao S. (London: Routledge) 202–216.

[B102] Wellman H. M. (1992). *The Child’s Theory of Mind.* Boston, MA: The MIT Press.

[B103] Wellman H. M. (2014). *Making minds: How Theory of Mind Develops.* Oxford: Oxford University Press.

[B104] White S. J. (1997). Empathy: a literature review and concept analysis. *J. Clin. Nurs.* 6 253–257. 10.1111/j.1365-2702.1997.tb00313.x 9274226

[B105] Wolf M. (2018). *Reader, Come Home.* New York, NY: HarperCollins Publishing.

[B106] Wynn K. Bloom P. Jordan A. Marshall J. Sheskin M. (2017). Not noble savages after all: limits to early altruism. *Curr. Dir. Psychol. Sci.* 27 3–8. 10.1177/0963721417734875 29713124 PMC5921922

[B107] Zaki J. (2014). Empathy: a motivated account. *Psychol. Bull.* 140:1608. 10.1037/a0037679 25347133

[B108] Zhao S. Unsworth L. (2016). “Touch design and narrative interpretation,” in *Apps, Technology and Younger Learners: International Evidence for Teaching* eds Kucirkova N. F. Falloon G. (London: Routledge) 89–102.

[B109] Zimbardo P. (2017). *Young Men and the Empathy Gap.* New York, NY: Psychology Today.

